# Effect of soybean ureases on seed germination and plant development

**DOI:** 10.1590/1678-4685-GMB-2016-0107

**Published:** 2017-03-02

**Authors:** Ciliana Rechenmacher, Beatriz Wiebke-Strohm, Luisa A. de Oliveira-Busatto, Joseph C. Polacco, Célia R. Carlini, Maria H. Bodanese-Zanettini

**Affiliations:** 1 Programa de Pós-Graduação em Genética e Biologia Molecular, Universidade Federal do Rio Grande do Sul (UFRGS), Porto Alegre, RS, Brazil; 2 Department of Biochemistry and Interdisciplinary Plant Group, University of Missouri, Columbia, MO, USA; 3 Departamento de Biofísica e Centro de Biotecnologia, Universidade Federal do Rio Grande do Sul (UFRGS), Porto Alegre, RS, Brazil; 4 Instituto do Cérebro (InsCer), Pontifícia Universidade Católica do Rio Grande do Sul (PUCRS), Porto Alegre, RS, Brazil

**Keywords:** Plant development, functional study, transgenic plants, mutants, urease

## Abstract

Urease catalyzes the hydrolysis of urea to ammonia and carbon dioxide. The ammonia (nitrogen (N) product of urease activity) is incorporated into organic compounds. Thus, urease is involved in N remobilization, as well as in primary N assimilation. Two urease isoforms have been described for soybean: the embryo-specific, encoded by the *Eu*1 gene, and the ubiquitous urease, encoded by *Eu*4. A third urease-encoding gene was recently identified, designated *Eu*5, which encodes the putative protein product SBU-III. The present study aimed to evaluate the contribution of soybean ureases to seed germination and plant development. Analyses were performed using *Eu*1/*Eu*4/*Eu*5-co-suppressed transgenic plants and mutants of the *Eu*1 and *Eu*4 urease structural genes, as well as a urease-null mutant (*eu3-a*) that activates neither the ubiquitous nor embryo-specific ureases. The co-suppressed plants presented a developmental delay during the first month after germination; shoots and roots were significantly smaller and lighter. Slower development was observed for the double *eu*1*-a*/*eu*4*-a* mutant and the *eu*3*-a* single mutant. The N content in transgenic plants was significantly lower than in non-transgenic plants. Among the mutants, *eu*3*-a* presented the lowest and *eu*1-a the highest N content. Altogether, these results indicate that increased ureolytic activity plays an important role in plant development.

## Introduction

Nitrogen (N) is the most limiting plant nutrient, possibly after fixed carbon, for plant growth and development (Marschner, 2012). Therefore, efficient mechanisms both to take up N in its various forms and to reallocate it are necessary for optimal N use efficiency (Witte, 2011). In plant cells, urea is an important internal and external source of N that must be converted to ammonia for N assimilation (Wang *et al*., 2008). In nature, two major biochemical processes lead to urea production: (1) arginase-catalyzed production of urea (and ornithine) from arginine (a major N storage form); (2) purine degradation to glyoxylate and urea. While arginases are active in plants, purine degradation exclusively through urea, though occurring in many bacteria, fungi and algae, does not occur in soybean and Arabidopsis. Rather, in these dicotyledonous plants, the purine degradation product allantoin, which contains the four ring N atoms of purine, is degraded to four ammonia molecules, bypassing a urea intermediate (summarized in Witte, 2011).

Nonetheless, plant assimilation of arginine-derived urea is important for efficient N use, both in mobilization of seed N reserves during germination, as well as in remobilization of N in senescing tissues. In soybean, arginine is the major amino acid repository of seed N (Micallef and Shelp, 1989), and one of the predominant amino acids in angiosperm seed protein in general (Van Etten *et al*., 1963). In soybean, arginase action during germination releases much urea, which is hydrolyzed by urease action (Goldraij and Polacco, 1999, 2000)

The importance of urease for recycling N was highlighted in aged *Arabidopsis thaliana* seeds that failed to germinate when urease was chemically inhibited, but could be rescued by an external N source (Zonia *et al*., 1995). And, according to Bohner *et al*. (2015) 13% of N exported out of senescing leaves of *A. thaliana* via the petiole is urea. Urease-negative soybean (mutants and nickel-deprived wildtype) accumulate urea in necrotic leaf tips (Stebbins *et al*., 1991) to levels approaching 2.5% dry weight (Eskew *et al*., 1983). We note that in Stebbins, (1991) and Eskew *et al*. (1983) available N was not limiting.

Urea can be hydrolyzed by two different enzymes: urease and an ATP (and biotin)-dependent urea carboxylase/allophanate hydrolase. The latter, found in some fungi, algae and at least one bacterium (Kanamori *et al*., 2004), has never been reported in plants. Rather, all plants appear to have a urease (Hogan *et al*., 1983 and our own observations). In soybean and Arabidopsis, urea nitrogen is only available after urea hydrolysis by urease (Goldraij *et al*., 2003; Witte, 2011; Polacco *et al*., 2013). Urease (EC 3.5.1.5) was the first identified nickel-dependent metalloenzyme (Dixon *et al*., 1975), and much has been learned of the construction and function of its metallocenter (Carter *et al*., 2009). Urease catalyzes the hydrolysis of urea to ammonia and carbon dioxide. In addition to plants, ureases are synthesized by bacteria, fungi and algae (Krajewska, 2009). The N product of urease activity - ammonia - is incorporated into organic compounds mainly by glutamine synthase activity (Mobley *et al*., 1995; Sirko and Brodzik, 2000). Thus, urease is involved in N remobilization, as well as in primary N assimilation (Cao *et al*., 2010).

In addition to the N assimilatory function of urease, plant ureases appear to have defensive roles against herbivore and fungal attack (Carlini and Ligabue-Braun, 2016). In soybean, three urease isoforms have been described. The ubiquitous urease, encoded by the *Eu*4 gene, is expressed at low levels in all tissues and is responsible for recycling both metabolically-derived and exogenous urea (Polacco *et al*., 1985; Torisky *et al.*, 1994; Goldraij *et al*., 2003). The embryo-specific urease, encoded by *Eu*1, is highly expressed in developing embryos and accumulates in mature seeds (Polacco and Havir, 1979; Polacco and Winkler, 1984; Polacco and Holland, 1993). A third urease-encoding gene was recently identified in the soybean genome (Polacco *et al*., 2011, 2013). This gene was designated *Eu*5, and its putative protein product was named SBU-III. *Eu*5 is expressed in the first stages of root development and during seed maturation. Its transcript levels are lower than those of the other two soybean urease isoforms (Wiebke-Strohm *et al*., 2016).

Urease-negative mutant soybean plants were examined to ascertain the role(s) of the urease isoforms. An embryo-specific urease null mutant (*eu1-a*) seems not to exhibit an altered physiology (Polacco *et al*., 2011). Ubiquitous urease missense mutants (e*u4-a* and e*u4-b*) produce an inactive protein and display no ureolytic activity in leaves, roots and hypocotyls (Torisky and Polacco, 1990; Stebbins *et al*., 1991; Witte *et al*., 2002, Goldraij *et al*., 2003; Polacco *et al*., 2011). e*u4* callus cultures cannot use 5 mM urea as N source, but are resistant to 50 mM urea in the presence of a standard NH_4_^+^ + NO_3_^-^ N source, and show growth responses contrary to those of *Eu4* cultures (Goldraij *et al*., 2003). The *eu*1*-a*/*eu*4*-a* double mutants were considered virtually devoid of ureolytic activity (Stebbins and Polacco, 1995; Goldraij *et al*., 2003).

A null mutant for the *Eu*3 gene, which encodes an accessory protein, UreG, necessary for urease activation, has also been characterized (Freyermuth *et al*., 2000). There is only a single copy of this gene in the soybean genome, and the deletion mutant *eu*3*-a* exhibits a complete loss of urease activity (Stebbins and Polacco, 1995; Polacco *et al*., 2011; Tezotto *et al*., 2016).

A previous study was performed by our team aiming to overexpress *Eu*4 in soybean plants. Unexpectedly, the transgenic plants exhibited co-suppression of the endogenous and the introduced *Eu4* transgene, resulting in decreased ureolytic activity (Wiebke-Strohm *et al*., 2012). As null mutants for the ubiquitous urease have not been obtained to date, the co-suppressed transgenic plants represent a powerful tool for functional gene studies. Here, we sought to determine the roles of urease in soybean development by elimination of all urease isoforms.

## Material and Methods

### Plant material and growth conditions

Homozygous *eu1-a, eu4-a*, *eu*1*-a*/*eu*4*-a* and *eu3-a* mutants have been described previously. All, except *eu*1*-a*, were recovered from EMS (ethyl methane sulfonate) mutagenesis of cv. Williams, and were subsequently outcrossed to Wiliams 82. The original *eu*1*-a* mutation was recovered from the “Itachi' landrace and introgressed into Williams by Dr Dick Bernard (University of Illinois-Champaign-Urbana) by five crosses. Thus, the genetic background of these mutants is the Williams (*eu*1*-a)* and Williams82 (*eu*4*-a*, *eu*1*-a*/*eu*4*-a* and *eu*3*-a*) cultivars. Williams and Williams82 are supposedly isogenic, except for a fungal resistance gene introgressed into Williams 82 (Bernard and Cremeens, 1988).

Two independent transgenic events (A3 and A8) of soybean cultivar IAS5 that presented co-suppression of *eu4* were obtained from bombarded embryogenic tissue. The vector used for transformation contained the *Eu*4 and the *gfp*-encoding sequences (Wiebke-Strohm *et al*., 2012). Plants derived from non-transgenic embryogenic tissues submitted to the same culture conditions were recovered and used as a control. Subsequent generations were obtained by self-fertilization of plants.

Transgenic seeds of A3 and A8 events (from T_1_, T_2_ and T_3_) were placed in Petri dishes containing sterile filter paper moistened with sterile distilled water for 24 h. Seeds expressing the *gfp* reporter were selected under blue light using a fluorescence stereomicroscope (Olympus^®^), equipped with a BP filter set with a 488 nm excitation filter and a 505-530 nm emission filter. GFP-positive and negative plants were also PCR-screened to confirm presence/absence of the transgene using the protocol described by Wiebke-Strohm *et al*. (2012). Positive transgenic, as well as non-transgenic seeds, were sown in organic soil and maintained in a greenhouse until maturity at FUNDACEP-CCGL (Cruz Alta, RS, Brazil) and supplemented with a nutrient solution containing either NO_3_ or NH_4_ (as N source).

For seed germination and developmental evaluation, GFP-positive transgenic (T_2_), mutants, IAS5 non-transgenic and Williams82 seeds were sown in pots containing vermiculite and maintained for one month in a growth chamber at 26 ± 1° C with a 16/8 h light/dark cycle at a light intensity of 22.5 μEm^-2^s^-1^. Plants were not supplemented with any nutrient solution during the first 30 days of development.

### RNA extraction, cDNA synthesis and quantitative real-time PCR (RT-qPCR)

Total RNA was extracted from roots with Trizol reagent (Invitrogen) and treated with DNase I (Invitrogen) according to the manufacturers' instructions. First-strand cDNAs were obtained using 1 μg of DNA-free RNA, M-MLV Reverse Transcriptase SystemTM (Invitrogen) and oligo(dT) primers.

RT-qPCR was performed on a StepOne Real-time Cycler^TM^ (Applied Biosystems). PCR-cycling conditions were implemented as described: 5 min at 94 °C, followed by 40 cycles of 10 s at 94 °C, 15 s at 60 °C and 15 s at 72 °C. A melting curve analysis was performed at the end of the PCR run, over the range of 55-99 °C, increasing the temperature stepwise by 0.1 °C every 1 s. Each 25-μL reaction comprised 12.5 μL cDNA (1:50 dilution), 1x PCR buffer (Invitrogen), 2.4 mM MgCl_2_, 0.024 mM dNTPs, 0.1 μM of each primer, 2.5 μL of SYBR-Green (1:100,000, Molecular Probes) and 0.03 U of Platinum Taq DNA Polymerase (5 U/μl, Invitrogen). All PCR assays were performed in technical quadruplicates and 10 biological samples. Reactions lacking cDNA were used as negative controls.

Transcript levels of the three urease-encoding genes were evaluated. The F-box protein and a metalloprotease were used as references for gene expression normalization (Jian *et al*,. 2008; Libault *et al*., 2008). Primer sequences are presented in Table 1. The expression data analyses were performed after comparative quantification of amplified products using the 2^-ΔΔCt^ method (Livak and Schmittgen, 2001).

**Table 1:** Primer set designed for RT-qPCR.

Target gene	Orientation	Primer sequence
*Eu*1 (embryo-specific urease)	Forward	5′-ACCAGTTTTGCAACCACCTT-3′
	Reverse	5′-AAGAACAAGAGCAGGGGAACT-3′
*Eu*4 (ubiquitous urease)	Forward	5′-TCACTGTGGACCCAGAAACA-3′
	Reverse	5′-CTTGCTTATTGTTTTTTGCCAAT-3′
*Eu*5 (urease III)	Forward	5′-GTCGAGTTGGAGAGGTCCTTTAT-3′
	Reverse	5′-GAGAAATGTCACATGCACACTG-3′
Metalloprotease	Forward	5′-ATGAATGACGGTTCCCATGTA-3′
	Reverse	5′-GGCATTAAGGCAGCTCACTCT-3′
FBox protein	Forward	5′-AGATAGGGAAATGTTGCAGGT-3′
	Reverse	5′-CTAATGGCAATTGCAGCTCTC-3′

### Ureolytic activity

Urease activity in transgenic (A3 and A8) and non-transgenic plants was determined with a urease indicator solution: 6 g urea, 10 mL cresol red (1 mg/mL ethanol), 10 mL KH_2_PO_4_/K_2_HPO_4_/EDTA (10 mM KPi/1 mM EDTA pH 7.0) and 1 mL sodium azide 20% (w/v) per liter (Meyer-Bothling and Polacco, 1987). Powdered leaves and roots ( ± 100 mg) of two-week old plants, were incubated in a 1 mL urease indicator solution for 24 h at 60 °C, as well as mature seed slices that were incubated for 20 min at room temperature. The *eu3-a* mutant was used as negative control. As urea is hydrolyzed by urease, the ammonia released increases the pH and turns to pink the initial yellow coloration of the cresol red pH indicator.

### Germination

Germination capacity of seeds (T_2_) from two transgenic events (A3 and A8) was compared with that of IAS5 non-transgenic seeds. For *eu1-a, eu*4*-a, eu*1*-a*/*eu*4*-a* and *eu*3*-a* mutant seeds germination capacity was compared with that of Williams82 non-mutant seeds. Seed germination, defined as radicle protrusion, was recorded over one month.

### Developmental pattern

Seven, 14, 21 and 30 days after sowing, seedlings from all genotypes were classified according to their developmental stage following the categories proposed by Neumaier *et al*. (2000): VE = emergence of cotyledons; VC = completely opened cotyledons; V1 = completely developed unifoliate leaf pair; V2 = completely developed first trifoliate leaf; V3 = completely developed second trifoliate leaf. Eighteen plants derived from each event (A3 and A8), 10 non-transgenic plants, 18 plants from each mutant and 10 non-mutant plants were observed. In addition, the dry matter and length of roots and shoots were also determined one month after sowing.

### Grain yield

The number of seeds produced per plant in three generations of transgenic (T_1_, T_2_ and T_3_) and non-transgenic plants were compared.

### Nitrogen content

Shoot nitrogen content (N mobilized from the cotyledons**)** was measured by the Kjeldahl method according to the methodology described by Tedesco *et al*. (1995) one month after sowing**.** This analysis evaluated 18 plants derived from each line A3 and A8 and 10 non-transgenic ones. Eighteen plants from each mutant (*eu*1*-a, eu*4*-a, eu*1*-a*/*eu*4*-a* and *eu*3*-a*) and non-mutant plants were also evaluated.

### Statistical analysis

A Student's *t*-test was used to compare the expression levels (RT-qPCR) of urease-encoding genes (*Eu*4*, Eu*1*, Eu*5) in roots of transgenic vs. non-transgenic plants and mutant vs. non-mutant plants.

In order to compare developmental stages, a score was attributed to each developmental category (VE = 1, VC = 2, V1 = 3, V2 = 4, V3 = 5). A generalized linear model for repeated measures was used to compare plant development among genotypes (IAS5 transgenic vs. IAS5 non-transgenic plants; *eu1-a* or *eu4-a* or *eu3-a* or *eu*1*-a*/*eu*4*-a* mutant vs. Williams82 non-mutant plants). ANOVA followed by Bonferroni's post hoc test were performed on dry matter, length and weight of roots and shoots data. A Student's *t*-test was carried out in order to compare the number of seeds produced per transgenic and non-transgenic plant in different generations (T_1_, T_2_ and T_3_). Data on shoot nitrogen content was compared among genotypes by ANOVA followed by Tukey's post hoc test. Analyses were performed using SPSS 18.0 software.

## Results

### Gene expression

**Figure 1:**
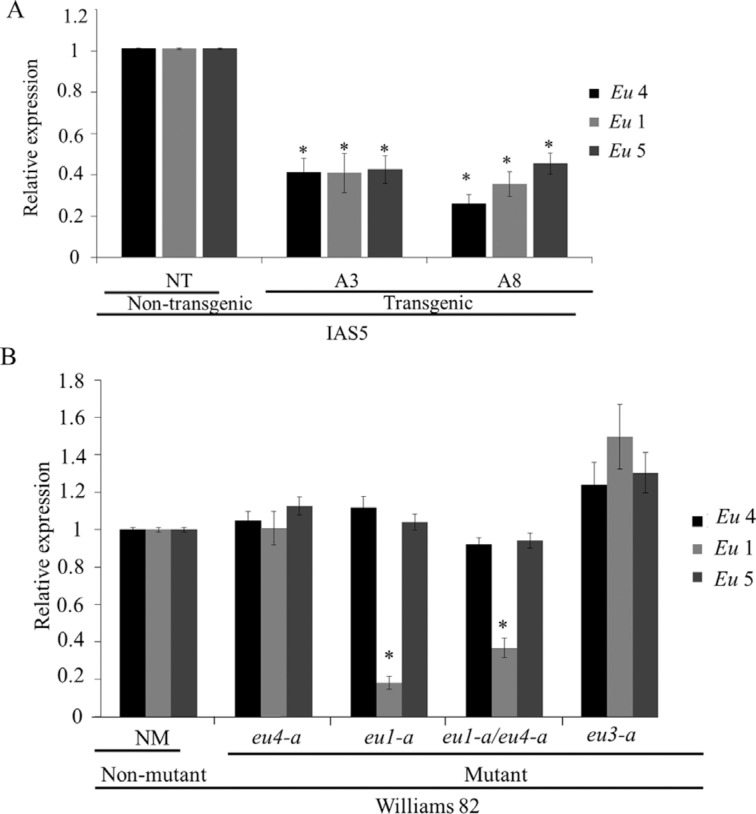
Transcript levels (RT-qPCR) of urease-encoding genes (*Eu*4, *Eu*1, *Eu*5) in roots of two-week-old plants. (A) Two independent transgenic events (A3 and A8) and non-transgenic plants (NT, control) from cv. IAS5. (B) Williams82 non-mutant (NM, control) and *eu4-a*, *eu1-a*, *eu1-a/eu4-a* and *eu3-a* mutants. The bars represent mean ± SD of two non-transgenic plants, 10 transgenic plants from each event, two non-mutant plants and 10 plants from each mutant. Transcripts level of *Eu*4, *Eu*1 and *Eu*5 detected in non-transgenic or non-mutant plants were used to normalize transcript accumulation in transgenic or mutant plants, respectively. F-Box and Metalloprotease reference genes were used as internal controls to normalize the amount of mRNA present in each sample. * indicates that the mean of each gene is significantly different from the control (*t* -test, p < 0.05).

Roots were chosen for gene expression analysis by RT-qPCR because *Eu*5 transcripts are mainly detected in this organ (Wiebke-Strohm *et al*., 2016). As expected, the progeny of transgenic plants showed lower *Eu*4 transcript levels than non-transgenic controls, suggesting that the co-suppressed phenotype was maintained. Additionally, it was observed that the other urease-encoding genes, *Eu*1 and *Eu*5, were also down-regulated (Figure 1A).

The transcript levels of all three urease-encoding genes in the mutants plants followed the predicted pattern: *eu*4*-a* and *eu3-a* displayed normal mRNA levels of the three genes; *eu*1*-a* and *eu*1*-a*/*eu*4*-a* presented lower levels of *Eu*1, but normal *Eu*4 and *Eu*5 expression levels (Figure1B). It is worth noting that although not affecting the mRNA expression levels, the *eu*4-*a* (and *eu*4-*b*) mutant produces a non-functional enzyme with a single amino acid replacement (Goldraij *et al*., 2003).

### Ureolytic activity

Ureolytic activity in transgenic and non-transgenic plants was evaluated with cresol red pH indicator by the seed chip assay of dried samples of leaves, roots and seeds. As expected, samples containing non-transgenic tissues showed pink coloration, indicative of urea hydrolysis. On the other hand, leaf and root samples of transgenic plants showed no observable color change even after 24 h incubation at 60 °C, indicating absence or drastic reduction of urease activity. Slices of mature seeds exhibited little or no activity. Very low urease activity was confirmed comparing transgenic with *eu3-a* seeds (used as a negative control) (Supplemental Figure S1).

Taken together, urease expression and activity assays indicate that transgenic plants have had all three urease-encoding genes silenced in all tissues, reinforcing the potential of these plants for functional studies.

### Germination

The germination rate of T_2_ seeds from the two independent transgenic events was evaluated and compared to that of non-transgenic seeds. No differences were detected, suggesting that the absence of all three ureases did not affect germination. The same result was observed on germination of the *eu1-a*, *eu4-a, eu1-a/eu4-a* and *eu3-a* mutants and non-mutant Williams82. Germination rates were higher than 90% for all genotypes (data not shown).

### Developmental pattern

**Figure 2:**
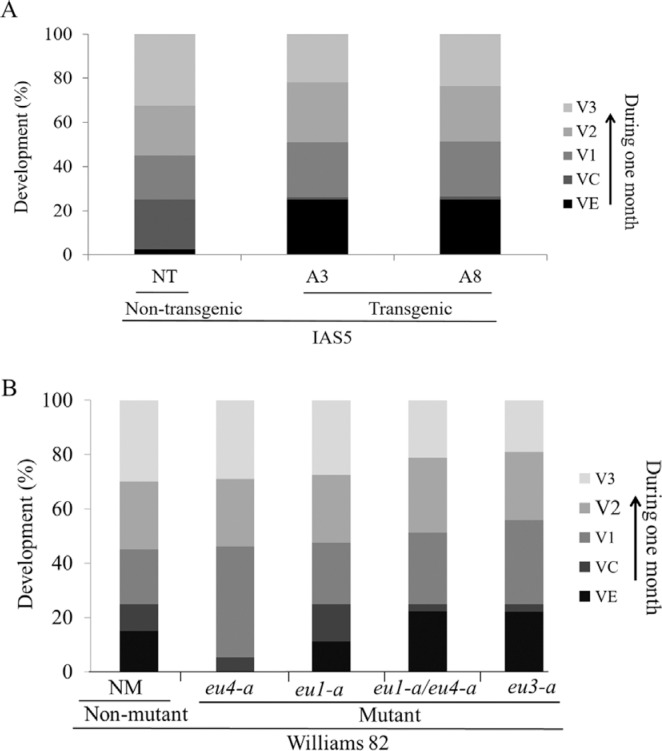
Plant developmental pattern over the first month after germination. (A) Transgenic and non-transgenic plants were evaluated: 18 plants derived from each event (A3 and A8) and 10 non-transgenic plants. (B) *eu4-a, eu1-a, eu1-a/eu4-a* and *eu3-a* mutant and non-mutant plants were evaluated: 18 plants from each mutant and 10 non-mutant plants. Plants were classified according categories: VE= emergence of cotyledons; VC= completely opened cotyledons; V1 = completely developed unifoliate leaf pair; V2= completely developed first trifoliate leaf; V3= completely developed second trifoliate leaf. A generalized linear model for repeated measures was used to compare the plant development among genotypes (p < 0.01).

**Figure 3:**
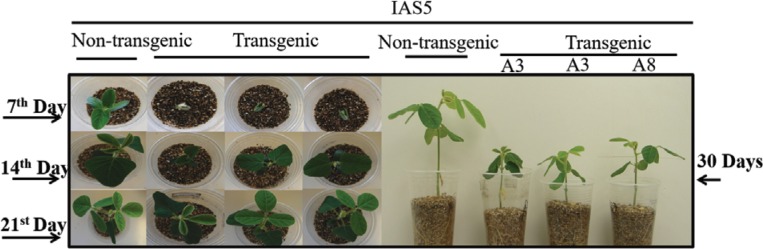
Transgenic and non-transgenic plants seven, 14, 21 and 30 days after germination. Transgenic plants and non-transgenic plants are from cv. IAS5.

Plant development was evaluated 7, 14, 21 and 30 days after sowing. T_2_ transgenic plants and non-transgenic plants, as well as *eu1-a*, *eu4-a, eu1-a/eu4-a* and *eu3-a* mutant and non-mutant plants were classified into developmental categories. Interaction among genotype, developmental categories and time-course was highly significant (p < 0.01). The two independent transgenic events showed a significant delay in development when compared with non-transgenic plants (Figure 2A and Figure 3). Size and dry weight of shoots and roots were significantly lower in transgenic plants when compared with non-transgenic (Table 2).

**Table 2:** Dry matter and length of roots and shoots one month after germination.

Genotype		Shoot		Root	
		length(cm)	weight(g)	length(cm)	weight(g)
IAS5	NT	12.58 a	0.63a	21.46a	0.37a
	A3	7.70b	0.29b	13.68b	0.21b
	A8	9.61b	0.39b	14.66b	0.28b
Williams 82	NM	12.57A	0.59 AB	55.65A	0.45AB
	*eu4-a*	13.65A	0.69A	47.97A	0.58A
	*eu1-a*	13.62 A	0.71A	52.37A	0.62A
	*eu1-a/eu4-a*	10.02B	0.51B	45.30AB	0.31B
	*eu3-a*	10.61B	0.47B	22.58B	0.23B

The developmental pattern of mutant plants was compared to non-mutants. The *eu1-a* single mutant showed a pattern similar to that of the non-mutant. The *eu4-a* mutant developed faster than control plants. Slower development was observed for the *eu1-a/eu4-a* double mutant and the *eu3-a* single mutant when compared to the non-mutant plants (Figure 2B). One month after germination, no differences were observed among the shoot and root sizes of *eu1-a* and *eu4-a* mutants and non-mutant plants. The shoot sizes of *eu1-a/eu4-a* double mutants and *eu3-a* single mutants were significantly smaller than those of the other genotypes. Regarding shoot and root weight, the *eu1-a/eu4-a* and *eu3-a* mutants were lighter than the other two mutants, but did not differ significantly from control. Total root length analysis showed that mutant *eu3-a* had the smallest root system (Table 2).

### Nitrogen content

**Figure 4:**
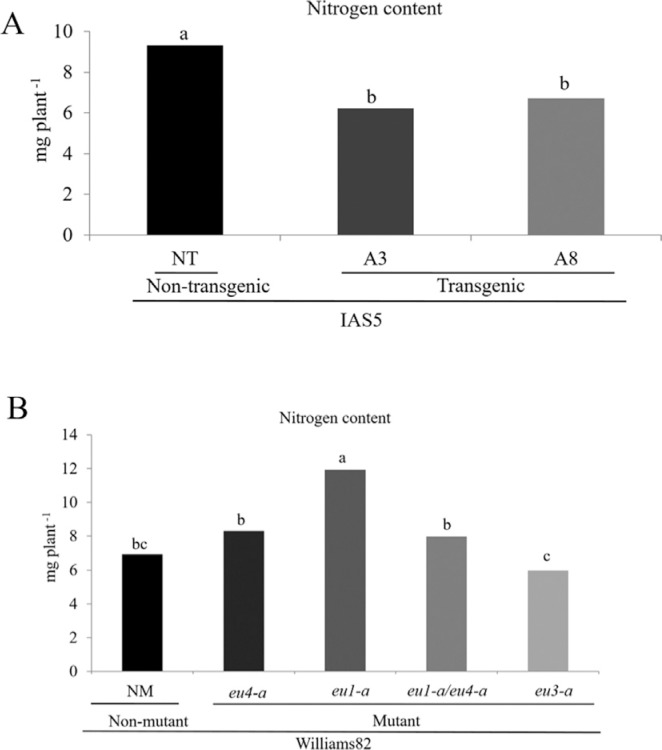
Nitrogen content rate in shoot of soybean at one month after germination. (A) Transgenic and non-transgenic plants were evaluated: 18 plants derived from each event (A3 and A8) and 10 non-transgenic plants. (B) *eu4-a, eu1-a, eu1-a/eu4-a* and *eu3-a* mutant and non-mutant plants were evaluated: 18 plants from each mutant and 10 non-mutant plants. ANOVA, p < 0.0001. Means followed by the same letter did not differ by Tukey's post hoc test.

The nitrogen content was measured in shoots of one-month-old plants. The N content in transgenic plants of the two independent events was significantly lower than that present in non-transgenic plants (Figure 4A). The comparison among mutants showed that *eu*3*-a* presented the lowest and *eu*1*-a* the highest N content (Figure 4B)

### Grain yield

**Figure 5:**
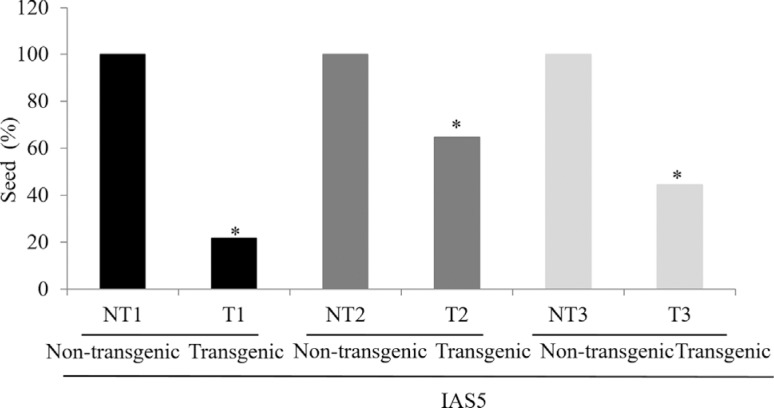
Relative percentage of seeds produced by transgenic plants in three generations (T_1_, T_2_ and T_3_). The mean number of seeds produced by non-transgenic plant was considered 100%. Transgenic plants and non-transgenic plants are from cv. IAS5. * indicates that the mean of transgenic seeds is significantly different from non-transgenic in each generation (*t*-test, p < 0.05).

The number of seeds produced by three generations of transgenic plants was compared with those produced by non-transgenic plants. A significantly lower number of seeds was obtained for transgenic plants (Figure 5).

## Discussion

The present study aimed to evaluate the contribution of soybean ureases to seed germination and plant development. Analyses were performed using co-suppressed transgenic plants and plants with mutations in urease-related genes. The transgenic plants were the progeny (T_2_) of two independent events in which the *Eu*4 gene was down-regulated as previously described (Wiebke-Strohm *et al*., 2012). Molecular analyses showed that *Eu*4 co-suppression was maintained in the transgenic progeny. In addition it was verified that transgenic plants exhibited very low transcript levels of the other two ureases encoded by the *Eu*1 and *Eu*5 genes. The phenomenon of co-suppression by transgenic DNA has been observed in many organisms, with the introduction of transgenic copies of a gene resulting in reduced expression of the transgene, as well as of the endogenous gene. This effect depends on the sequence identity between transgene and endogenous gene (Ketting and Plasterk, 2000).

Soybean plants with mutations in urease genes were also evaluated confirming the expected expression pattern. Normal transcript levels of all three urease-encoding genes were detected for *eu4-a* and *eu3-a* mutants. This result is consistent with the *eu*4*-a* allele encoding G468E missense-altered ubiquitous urease (Goldraij *et al*., 2003). *Eu*3 is the only UreG-encoding gene in the soybean genome. UreG is essential for urease activation, and the *eu*3*-a* mutant presents a complete loss of urease activity (Freyermuth *et al*., 2000), consistent with a > 90% deletion of the UreG ORF (Tezotto *et al*., 2016). The *eu*3*-a* mutation did not alter *Eu*4, *Eu*1 and *Eu*5 expression levels, indicating a lack of feedback control on urease structural gene transcription by apo-urease(s). The mutants *eu1-a* and *eu1-a/eu4-a* exhibited similar expression patterns: lower *Eu*1, but normal *Eu*4 and *Eu*5 transcript levels. Since *eu1-a* is a null mutant (Polacco and Holland, 1993), low levels of *Eu*1 transcripts are expected, and indeed, Torisky *et al*. (1994) employing *Eu*1-specific PCR primers, recovered no detectable product from *eu*1*-a* embryo cDNA.

Ureolytic activity in transgenic and non-transgenic plants was evaluated by the seed chip assay. As expected, leaf and root samples of transgenic plants showed no color change even after 24 h incubation. The urease activity level can be inferred based on the time change “yellow to pink” on the seed chip assay (Polacco *et al*., 2011). According to the authors, 0.2% normal urease specific activity requires 10 hours for changing the solution color. When the activity decreases to 0.15%, the time required for solution color change is 48 hours. Based on these data, we conclude that the urease activity in leaves and roots of transgenic plants was absent or less than 0.2%, since no change in color was observed after a 24 hours incubation. The reaction catalyzed by urease is essential to allow most organisms (those lacking urea carboxylase) to use external or internally generated urea as a nitrogen source (Mobley and Hausinger, 1989; Mobley *et al*., 1995).

It has been demonstrated that aged *A. thaliana* seeds fail to germinate when urease was chemically inhibited, but seed viability could be rescued by an external N source (Zonia *et al*., 1995). In the present study no differences were detected in germination rates of transgenic and mutant seeds. However, it is important to highlight that soybean seeds were not aged and have a much higher protein content than *A. thaliana* seeds.

An association between urease activity and developmental pattern was observed. Transgenic plants, as well as *eu3-a* and the double *eu1-a/eu4-a* mutants, showed a delay over the first month after sowing. The delay in development was maintained even in adult transgenic plants and may be the cause of lower seed production. In *A. thaliana*, both urease transcripts and ureolytic activity increased after germination, especially in 8/9-day-old wild-type seedlings (Zonia *et al*., 1995). Embryo-specific urease (*Eu*1) activity in young soybean plants was also observed by Torisky and Polacco, (1990). Similarly, high transcript level of *Eu*4 and moderate transcript levels of *Eu*1 and *Eu*5 were detected in soybean seeds one day after dormancy break (Wiebke-Strohm *et al*., 2016). Taken together, these results indicate that increased urease content and/or ureolytic activity play a role in early stages of plant development.

A role in making nitrogen available during plant development has been attributed to soybean ubiquitous urease due to its catalytic activity and tissue distribution (Stebbins *et al*., 1991). However, our results indicate that *Eu*1 and *Eu*5 have a contribution in the developmental process as well. This is supported by the finding that transgenic A3 and A8, mutant *eu1-a/eu4-a* and *eu3-a* plants showed a delay in the first developmental stages. In transgenic plants, the impairment in development was confirmed by the significant reduction in size and weight of roots and shoots one month after germination. These data are consistent with significantly lower nitrogen content detected in transgenic plants. Regarding mutant plants, *eu3-a* tends to be smaller and lighter than the other genotypes, although significant differences were only detected for roots and shoots size. The reduction in dry matter is also reflected in the lowest nitrogen content. An unexpected result was the significantly higher nitrogen content present in *eu1-a*. The differences in nitrogen content might be due to differences in mutant genetic backgrounds. Williams is the background for *eu*1*-a*, while Williams82 is the one for the other mutants. In our experiment, non-mutant Williams82 was used as control.

Based on bioinformatics analyses, *Eu*5 has been suggested not to be a functional ureolytic enzyme due to a number of mutations, including deletions (Witte, 2011). However, according to our results the product of this gene might be involved in plant development. Further studies are necessary to elucidate whether the ureolytic activity and/or other non-enzymatic property of ureases are involved on plant development.

## Supplementary Material

The following online material is available for this article:

Figure S1 - Ureolytic activity in transgenic and non-transgenic plants.
