# Supplementary material for: Effect of soybean ureases on seed germination and plant development
Source: Genet Mol Biol. 2017 Mar 2;40(1 Suppl 1):209–16. doi: 10.1590/1678-4685-GMB-2016-0107 (PMC5452136; doi:10.1590/1678-4685-GMB-2016-0107)
Supplement: Supplementary file 1 [file 1415-4757-gmb-1678-4685-GMB-2016-0107-Suppl01.pdf]

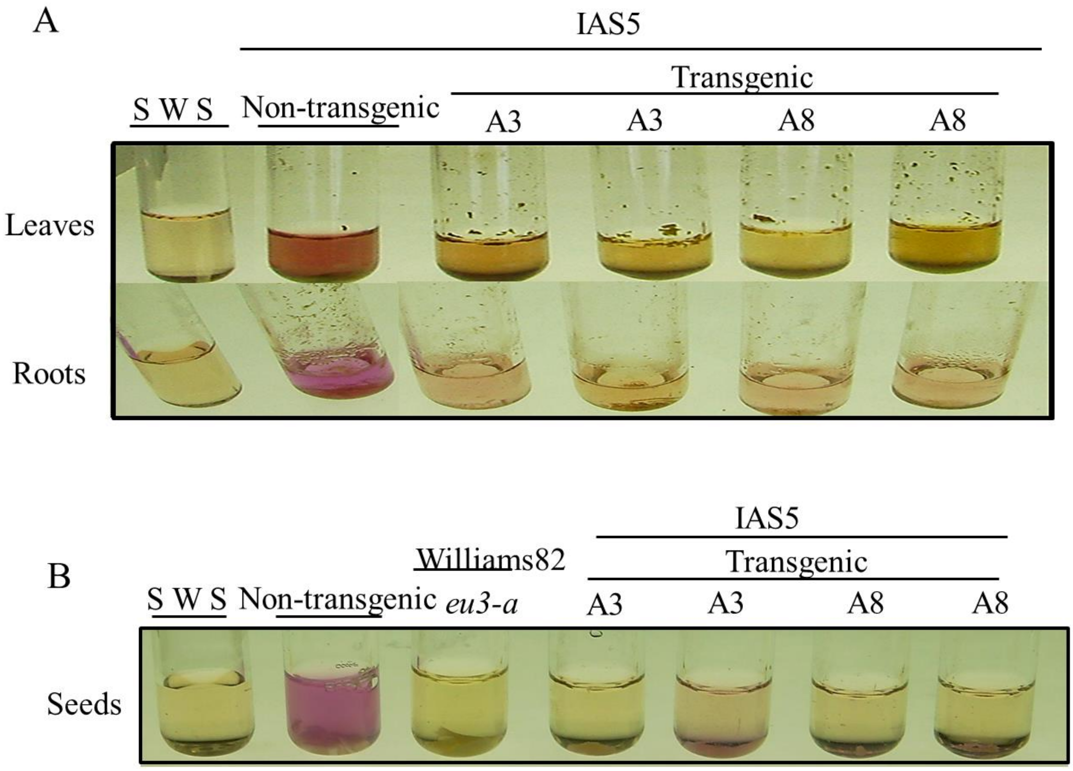

Figure S1- Ureolytic activity in transgenic and non-transgenic plants. (A) powdered leaves and roots of two-week old plants (B) slices of mature seeds were incubated in a pH-indicator reagent containing cresol red and weakly buffered 10 mM urea. As the ureolytic activity proceeds, the released NH_4^+ increases the pH, turning the solution from yellow to pinkish. SWS = solution without sample; non-transgenic plant from cv. IAS5; *eu3-a* mutant (used as negative control) and two transgenic plants from each event (A3 and A8).
